# Cervical Fractures: Does Injury Level Impact the Incidence of Dysphagia in Elderly Patients?

**DOI:** 10.3390/geriatrics2030021

**Published:** 2017-07-12

**Authors:** Jill Pattison, Michelle Kincaid, Urmil Pandya

**Affiliations:** 1 Heritage College of Osteopathic Medicine, Ohio University, Athens, OH 45701, USA; pattisonjill@gmail.com; 2 Trauma Services, Grant Medical Center, 111 South Grant Avenue, Columbus, OH 43215, USA; michelle.kincaid@ohiohealth.com

**Keywords:** dysphagia, geriatrics, cervical fracture

## Abstract

Dysphagia is common in the elderly with significant consequences such as aspiration and malnutrition. This study seeks to investigate oropharyngeal dysphagia in elderly patients with cervical fractures and determine whether the level of cervical fracture impacts the incidence of swallowing dysfunction. Records of trauma patients ≥65 admitted with cervical fractures over a 76-month period to a level 1 trauma center were reviewed. History of dysphagia, stroke, tracheostomy or spinal cord injury were excluded criteria, leaving 161 patients for analysis. Evaluation of swallowing function was performed to identify dysphagia and variables were analyzed. A total of 161 patients met inclusion criteria and 42 (26.1%) had dysphagia. Patients with dysphagia were older (84.1 ± 8.93 vs. 79.9 ± 8.48, *p* = 0.006), had higher hospital length of stay (9.0 ± 4.48 vs. 4.6 ± 3.30, *p* = <0.0001), and were more likely to have intensive care unit days (52.4% vs. 21.8%, *p* = 0.0002). Non-operatively-managed patients with C1 fractures were more likely to have dysphagia than patients without C1 fractures (29.2% vs. 7.1%, *p* = 0.0008). After regression analysis, C1 fracture increased the likelihood of dysphagia by four times (OR = 4.0; 95% CI 1.2–13.0). Oropharyngeal dysphagia is common in elderly patients with cervical fracture. Non-operatively-managed patients with C1 fractures are at increased risk and may benefit from more vigorous surveillance.

## 1. Introduction

Oropharyngeal dysphagia (OD), often referred to as swallowing dysfunction, is a prevalent condition in the geriatric population, affecting up to 13% of all people aged 65 and older [1,2]. Significant adverse consequences such as an increased risk for malnutrition, aspiration, and pulmonary complications [1,3,4,5,6] make recognition and management of this condition important in order to optimize clinical outcomes. Though medical risk factors such as stroke and neurologic disorders have been described in the literature [7,8,9,10], few studies have specifically examined the geriatric trauma population.

Cervical spine fractures are among the most common injuries sustained by the elderly and can result from seemingly low mechanism injuries such as ground level falls [11,12,13]. In geriatric patients, injuries located higher on the cervical spinal column are more frequent than lower injuries, with odontoid fractures being the most common in this population [11]. Management of cervical fractures can range from surgical stabilization to neck immobilization with cervical collars or halo braces, each of which can theoretically alter the physiology of swallowing. Though several previous studies have described an association between cervical spinal cord injury and OD [14,15,16,17,18], few have examined the effect of cervical fractures without spinal cord injury in the elderly.

The purpose of this study was to investigate the prevalence and risk factors for OD in geriatric patients who sustain cervical spine fractures without spinal cord injury. In addition, we sought to more closely examine the effect of the level of spinal column fracture on the rate of OD. We hypothesized that increased rates of swallowing dysfunction would be present with higher level cervical fractures.

## 2. Methods

After approval from the institutional review board at our level 1 trauma center in Columbus, Ohio, the trauma registry was queried for all trauma patients age >65 years with a diagnosis of cervical spine fracture admitted from January 2008 to April 2014. Patients with a previous history of dysphagia, stroke, or those with tracheostomy placement during the hospitalization were excluded. In addition, patients with the diagnosis of spinal cord injury were also excluded.

All patients were routinely assessed by nursing staff for signs or symptoms of swallowing dysfunction. Those found to have evidence or concern for swallowing dysfunction underwent a more formal swallowing evaluation with speech therapy consultation. Dysphagia was defined as any recommended restriction or diet modification as a result of speech therapy assessment. 

Demographic data and outcomes were collected and analyzed. Variables of interest included age, injury severity score, mechanism of injury, type of treatment, gender, hospital length of stay, intensive care unit days, vent days, mortality, presence of dysphagia and level of cervical spine fracture.

Descriptive statistics were reported as mean ± standard deviation for continuous variables, and frequencies with percentages for dichotomous or categorical variables. Percentages were compared between independent groups using chi-square tests. Where informative, odds ratios were reported, along with the corresponding 95% confidence interval. Means for continuous variables were compared between independent groups using *t*-tests (or, in the case of non-normal variables, Wilcoxon two-sample tests). Dichotomous outcomes were modeled using logistic regression.

## 3. Results

A total of 217 patients >65 years of age with cervical spine fracture were initially identified. Patients who had a tracheostomy, history of stroke or dysphagia, or spinal cord injury were excluded leaving 161 patients that met final inclusion criteria. Demographic data is shown in Table 1. The mean age of the cohort was 80.97 ± 8.78 with an average injury severity score (ISS) of 8.98 ± 3.47. The majority (76.4%) of patients were managed non-operatively while 23.6% were treated with surgical intervention. The most common level of fracture was C2 (72%) followed by C1 (36.6%). Fall was the most common mechanism of injury.

OD was present in 42 patients (26.1%) versus 119 (73.9%) patients without OD. Characteristics of the OD group were compared to the non OD group (Table 2). The mean age was significantly higher in the OD group. The incidence of OD was greatest higher age groups of 85 to 90 and greater than 90 (Table 3). Patients over the age of 85 had a significantly higher rate of OD (40.4% versus 19.3%, *p* = 0.004). Injury severity score was significantly higher in the OD group. Longer hospital length of stay, higher rate of intensive care unit (ICU) stay, and higher rate of mechanical ventilation were all associated with OD. Patients with OD were more like to be surgically managed (54.8% vs. 12.6%, *p* = <0.0001). Fall prior to admission as the mechanism of injury was significantly more likely in the OD group. There was no significant difference in the distribution of the level of cervical spine fracture between groups.

Subgroup analysis of patients who were non-operatively managed was performed. Patients with OD were compared to patients without (Table 4). Age and hospital length of stay remained significantly higher in the OD group. Patients with OD were significantly more likely to have a C1 fracture than those without OD and 29.2% of all non-operatively-managed patients with C1 fractures developed OD. After using regression analysis to control for age, hospital length of stay, and any ICU days, C1 fracture increased the likelihood of OD by four times (OR = 4.0; 95% CI 1.2–13.0).

## 4. Discussion

Difficulty in transferring a bolus from the mouth to the esophagus is often referred to as oropharyngeal dysphagia or swallowing dysfunction [19,20,21]. Swallowing is a complex process requiring coordination as well as multiple interacting components that can include up to 40 different muscles [22]. Impairment of bolus transit by structural abnormalities is a potential contributor to OD [19]. It has been shown that that iatrogenic causes such as intubation, tracheostomy, surgery, and radiation can lead to OD [20,23] In much the same way, it is possible that the presence of cervical fractures can produce subtle alterations in neck positioning, anatomy, or edema of the soft tissues which may adversely affect the swallowing mechanism in these patients.

Some estimates of OD suggest rates as high as 30 to 40% in the independently living geriatric population [19,24]. Hospitalization to a geriatric acute care center and institutionalization increase the prevalence further with rates of 44% [19,25,26] and 60% [19,27] respectively being reported. Over the next several decades, the proportion of elderly people in the United States is expected to climb with projections of 90 million people 65 years and older by the year 2050 [28]. In addition, increasingly active lifestyles have resulted in larger numbers of geriatric patients presenting with injury [12,13].

With a constantly aging population and larger proportion of geriatric patients making up the trauma population, there has been relatively little focus on OD as a problem for injured elderly patients. Cervical spine fractures are among the most common injuries sustained by the elderly [11,12,13]. Our study is unique in that it focuses on OD in the population of elderly trauma patients with cervical spine fractures.

Several previous studies have reported that cervical spinal cord injuries are associated with an increased incidence of dysphagia with rates from 17 to 41% [14,15,16,17,18]. To our knowledge, only one of these studies described a sub group of patients with cervical spine fractures who did not suffer from a spinal cord injury. Lee et al. found that 81% of the patients with cervical spine fracture that developed OD, did not have a spinal cord injury. One of the focuses of our study was to more closely examine patients with cervical fracture who did not sustain a spinal cord injury. Though Lee et al. did examine the geriatric population, they did not report on the rate of OD in this group that had cervical spine fracture without spinal cord injury. The incidence of OD in our cervical fracture geriatric patient population was 26.1%. This supports the fact that elderly patients with cervical fractures without spinal cord injury still have a high incidence of OD.

Well established risk factors for OD in the literature include the presence of a tracheostomy [11,16,18,28,29,30,31], stroke history [7,8,9,10], and as mentioned earlier, spinal cord injury [11,15,16,17,18]. By excluding patients with any of these preexisting risk factors, we hoped to obtain a more precise picture of what influences the rate of OD in the geriatric cervical fracture population. The previously mentioned analysis by Lee et al. reported an overall 24% rate of OD for patients of all ages with cervical injuries without spinal cord injuries. After eliminating the previously mentioned high risk predisposing factors, one would expect our rate may be lower, however, it was similar at 26.1%. The fact that we focused on elderly patients is the likely reasoning for this finding, reinforcing that age is a significant risk factor in and of itself for developing OD.

Patients with dysphagia were significantly older than those without. These finding corroborate those from other recent studies [11]. In our population, the incidence of dysphagia seemed to increase after the age of 85. Patients over the age of 85 had a rate of 40.4% versus 19.3% (*p* = 0.004) for patients younger than 85. The concept of OD as a geriatric syndrome that increases with age is apparent in our study group of cervical spine fracture patients as well.

There remains significant uncertainty as to the most optimal method of management of cervical spine fractures in the elderly. The risk of perioperative complications with surgical management must be weighed against the possibility of inadequate healing and prolonged immobilization that can occur with conservative management. Though studies have reported similar complication rates, the optimal treatment is still unclear [32,33,34]. Surgical management in our study population was associated with a significantly higher rate of OD than non-operative management. Post-operative swelling, increased likelihood of ventilator days, and higher injury severity may all have been contributory to this finding. This suggests that the increased risk of OD should be taken in to consideration when making the clinical decision to surgically manage cervical spine fractures in the elderly.

To our knowledge, no previous studies have assessed the effect that the level of cervical fracture has on OD rates. High cervical fractures are more common in the geriatric population with C2 fractures being the most frequent [11]. This was true in our population with C1 and C2 fractures being the most commonly sustained. We found no association between level of fracture and OD for the overall study population, however, subgroup analysis of patients non-operatively managed did show an association between C1 fracture and OD. After regression analysis, patients with C1 fractures were four times more likely to have OD. Of all patients with C1 fractures managed non-operatively, 29.2% had OD. When analyzing patients managed with c-collar alone, C1 fracture remained a significant predictor of OD. The reason for this correlation cannot be determined from this investigation, but future study to validate and determine why C1 fractures predict OD may be useful.

Though these findings should be validated in future study, our results suggest that geriatric patients with C1 fractures should be more aggressively screened for OD. The findings emphasize the fact that cervical immobilization with c collars may be detrimental to certain subsets of patients. Suboptimal neck positioning and post traumatic edema may contribute to anatomic alterations resulting in OD. Cervical bracing has been shown to alter the physiology of swallowing even in healthy adult patients [35]. Our study group included six patients who were non-operatively managed and did not require a collar for immobilization. None of these six patients developed OD. The results lack the statistical power to make any definitive conclusions, but a future study examining the effect of a collar versus no collar could provide more definitive results. Whether potential solutions such as collar removal at the time of feeding could help alleviate OD are areas that should be investigated in the future.

In the general population, literature has identified fall as a risk factor for the development of OD [21]. The most common mechanism of injury in the elderly is ground level falls [36,37] and this was also true in our patient population. Patients who fell were significantly more likely than those involved in motor vehicle collision (MVC) or other mechanisms to develop OD. It is possible that patients who fall are more likely have many of the predisposing characteristics such as functional and cognitive dependency that patients susceptible to OD have.

As with any retrospective study, this report had its limitations. The sample size limited statistical power for analysis of smaller subgroups within the population and could have impacted some of the results. Of note, we identified OD by systematically having a bedside nursing evaluation, followed by formal speech therapy consultation in indicated cases. Since not every patient was evaluated with a formal speech therapy consultation, it is possible that clinically borderline OD could have been missed in certain cases and led to some underestimation of the true incidence. Finally, discharge disposition to home versus nursing or rehab facility would be worth exploring in future study.

In conclusion, our results suggest that OD is common in geriatric trauma patients with cervical spine fracture. Age, increased injury severity score, hospital length of stay, vent days, and surgical management are all associated with an increased incidence. Geriatric patients with C1 fractures who are non-operatively managed may benefit from more aggressive screening and treatment to detect and manage OD.

## Figures and Tables

**Table 1:** Overall Demographics.

Characteristic	N
Total patients	161
Age	81.0 (8.78)
Injury severity score	9.0 (3.47)
Gender male	76 (47.2)
Total Hospital Length of Stay	5.8 (4.11)
Any intensive care unit days	48 (29.8)
Any vent days	19 (11.8)
Mortality	10 (6.21)
Management	
Surgical	38 (23.6)
C-collar	109 (67.7)
Halo	8 (4.97)
None	6 (3.73)
Level of Fracture	
C1	59 (36.6)
C2	116 (72.0)
C3	18 (11.2)
C4	20 (12.4)
C5	29 (18.0)
C6	43 (26.7)
C7	46 (28.6)
Mechanism of Injury	
Fall	131 (81.4)
Motor vehicle collision	24 (14.9)
Other	6 (3.73)
Dysphagia Y/N	42 (26.1)

**Table 2:** Comparison of outcomes dysphagia vs. no dysphagia.

Characteristic	Dysphagia	No Dysphagia	*p*-Value, Odds Ratio
Total patients	42	119	
Age	84.1 (8.93)	79.9 (8.48)	0.006
ISS	10.4 (3.70)	8.5 (3.25)	0.001
Gender male	20 (47.6)	56 (47.1)	0.95
Hospital LOS	9.0 (4.48)	4.6 (3.30)	<0.0001
Any ICU days	22 (52.4)	26 (21.8)	0.0002
Any vent days	10 (23.8)	9 (5.59)	0.005
Mortality	4 (9.52)	6 (14.3)	0.3
Management			
Surgical	23 (54.8)	15 (12.6)	<0.0001, OR = 8.4 (3.7, 18.9)
C-collar	16 (38.1)	93 (78.2)	<0.0001, OR = 5.8 (2.7, 12.4)
Halo	3 (7.14)	5 (4.20)	0.45
None	0 (0)	6 (5.04)	0.14
Level of Fracture			
C1	18 (42.9)	41 (34.5)	0.33
C2	35 (83.3)	81 (68.1)	0.058
C3	4 (9.52)	14 (11.8)	0.69
C4	3 (7.14)	17 (14.3)	0.23
C5	9 (21.4)	20 (16.8)	0.50
C6	13 (31.0)	30 (25.2)	0.47
C7	14 (33.3)	32 (26.9)	0.43
Mechanism of Injury			
Fall	40 (95.2)	91 (76.5)	0.007, OR = 6.2 (1.4, 27.1)
MVC	2 (4.76)	22 (18.5)	0.03, OR = 4.5 (1.0, 20.2)
Other	0 (0)	6 (5.04)	0.14

**Table 3:** Incidence of oropharyngeal dysphagia (OD) by age group.

Age Group	*n*	Total with OD
65–70	21	4 (19.0%)
70–75	25	5 (20.0%)
75–80	29	5 (17.2%)
80–85	34	7 (20.6%)
85–90	25	10 (40.0%)
>90	27	11 (40.7%)

**Table 4:** Comparison of dysphagia by level of C spine fracture in a non-operative population.

	OD (*n* = 19)	Non OD (*n* = 104)	*p*-Value
Age, mean (SD)	86.3 (8.01)	80.6 (8.76)	**0.007**
ISS, mean (SD)	9.1 (3.52)	8.4 (3.53)	0.46
Gender male, N (%)	11 (57.9%)	47 (45.2%)	0.31
Hospital LOS, mean (SD)	7.4 (4.23)	4.3 (4.21)	**0.016**
Any ICU days, N (%)	9 (47.4%)	21 (20.2%)	**0.01**
Any vent days, N (%)	2 (10.5%)	7 (6.7%)	0.56
Mortality, N (%)	3 (15.8%)	6 (5.8%)	0.12
C1 fx, N (%)	14 (73.7%)	34 (32.7%)	**0.0008**
C2 fx, N (%)	14 (73.7%)	68 (65.4%)	0.48
C3 fx, N (%)	3 (15.8%)	14 (13.5%)	0.99
C4 fx, N (%)	2 (10.5%)	16 (15.4%)	0.58
C5 fx, N (%)	3 (15.8%)	19 (18.3%)	0.80
C6 fx, N (%)	6 (31.6%)	25 (24.0%)	0.49
C7 fx, N (%)	6 (31.6%)	28 (26.9%)	0.68
